# The efficacy of Tuina for asthma: A protocol for a systematic review and meta-analysis

**DOI:** 10.1097/MD.0000000000023912

**Published:** 2020-12-24

**Authors:** Changhong Wang, Yong Jiang, Zhipeng Fan, Mao Zhao, Yuchang Jiang, Zhaodi Wang, Zhaoxing Chen

**Affiliations:** School of Basic Medicine, Chengdu University of Traditional Chinese Medicine, Chengdu, China.

**Keywords:** asthma, meta-analysis, protocol, systematic review, tuina

## Abstract

**Background:**

Asthma is one of the most common chronic diseases in the world, with ∼100 million asthma patients worldwide. China has become one of the countries with the highest asthma death rate in the world. Asthma is a chronic airway inflammatory disease. Patients with this disease may have symptoms such as cough, wheezing, and difficulty breathing. For many years, Western medicine has mainly used anti-inflammatory, anti-bronchial spasm, asthma, cough, and oxygen to treat this disease, but the effect is not good. Tuina is a common treatment for asthma in China. But at present, there is no systematic evaluation report on its therapeutic effectiveness and safety. This protocol aims to reveal the efficacy and safety of Tuina for treating asthma.

**Methods:**

The following databases will be searched by electronic methods: PubMed, EBASE, WHO International Clinical Trials Registry Platform, Embase, the Chinese Biomedical Literature Database (CBM), Wan-fang Data (WANFANG), the China National Knowledge Infrastructure (CNKI), and other sources from inception to November 2020. Bias risk, subgroup analysis, data synthesis, and meta-analyses will be assessed with RevMan V.5.3 software if the data is met inclusion conditions.

**Results:**

This study will present a quality evidence of Tuina for the treatment of astma patients.

**Conclusion:**

The systematic review will present reliable evidence to judge whether or not Tuina is a safe and effective intervention for asthma patients. International Platform of Registered Systematic Review and Meta-Analysis Protocols (INPLASY) registration number: INPLASY2020110100.

## 1 Introduction

Asthma is a major noncommunicable disease characterized by recurrent attacks of breathlessness and wheezing, which vary in severity and frequency from person to person.^[1]^ Symptoms may occur several times in a day or week in affected individuals, and for some people become worse during physical activity or at night.^[2]^ During an asthma attack, the lining of the bronchial tubes swell, causing the airways to narrow and reducing the flow of air into and out of the lungs.^[3]^ Recurrent asthma symptoms frequently cause sleeplessness, daytime fatigue, reduced activity levels and school and work absenteeism.^[4]^ asthma has a relatively low fatality rate compared to other chronic diseases.^[5]^ Tuina is the use of a certain part of the hand or limb by a doctor to act on a certain part of the patient to press, push, grasp, roll, pinch, etc, which could produce biological effect, improve the corresponding clinical symptoms.^[6]^ The disease seriously affects the quality of daily life of patients and imposes a huge burden on society and families.^[7]^ Serious respiratory illness may occur if not treated promptly or treated improperly, and severe cases may die as the disease progresses.^[8]^ In modern medicine, the cause of bronchial asthma is due to its chronic inflammatory response, and it should be adhered to the principle of symptomatic treatment and anti-inflammatory and antispasmodic treatment.^[9]^ Although it has certain therapeutic effects, it is easy to recurrent and the curative effect is greatly reduced.^[10]^

Studies have shown that Tuina can increase the concentration of Ca2+ in immune system mast cells, it can enhance immune function, unblocking, and collateral meridians activate Qi and blood, and improve the follow of Qi. It has been widely used in China and many other countries.^[11]^ However, at present, there is no systematic evaluation report on the efficacy and safety of Tuina in the treatment of shoulder periarthritis.^[12]^ The purpose of this article is to systematically review and analyze the relevant literature, provide effective, and safe evidence.

Therefore, we hope to evaluate the efficacy and safety of acupuncture in the treatment of asthma through meta-analysis, and provide a sufficient basis for its clinical application.

## 2 Study aim

The aim of the systematic review and meta-analysis was to systematically evaluate the efficacy and safety of Tuina mediated therapy for the treatment of advanced asthma.

## 3 Methods

This review protocol is registered in the International Platform of Registered Systematic Review and Mate-Analysis Protocols (INPLASY). The registration number was INPLASY2020110100 (DOI number is 10.37766/inplasy2020.11.0100). The systematic review will be conduct by Cochrane Handbook for Systematic Reviews of Interventions and reported according to preferred reporting items for the Systematic Review and Meta-Analysis Protocols (PRISMA-P) guidelines. The ethical approval or informed consent was not required in this study because it belongs to secondary research which based on some previously published data.

### 3.1 Dissemination plan

We will disseminate the results of this systematic review by publishing the manuscript in a peer-reviewed journal or presenting the finding at a relevant conference.

### 3.2 Eligibility criteria

#### 3.2.1 Study designs to be included

We will only include randomized controlled trial (RCTs), non-RCTs, quasi-RCTs, reviews, and other types of studies will be excluded.

#### 3.2.2 Participant or population

This review includes diarrhea patients regardless of race, region, sex, and the phase of asthma.

#### 3.2.3 Intervention

The main intervention is Tuina. The duration and frequency of Tuina are not limited.

#### 3.2.4 Comparator

There is no exclusion based on comparator method for this review, and the patients could be treated with any forms of control group.

#### 3.2.5 Type of outcome

##### 3.2.5.1 Main outcome(s)

The main criteria are:

1. Symptoms and signs disappear;
2. No significant wheezing after the event;
3. Stop the drug for 3 months without attack;
4. Film degree exam;
5. Lung ventilation

##### 3.2.5.2 Additional outcome(s)

Secondary assessment criteria include Lung sounds and cough disappears. At the same time, close attention should be paid to whether adverse reactions or adverse events occur during the experiment to comprehensively evaluate the clinical efficacy and safety of acupuncture in the treatment of asthma.

### 3.3 Information sources and search strategy

Four English electronic databases including Cochrane Library, PubMed, Web of Science, Excerpt Medical Database, four Chinese electronic databases include Chinese Biomedical Literature Database, China National Knowledge Infrastructure, China Scientific Journal Database, and Wanfang and WHO International Clinical Trials Registry Platform will be systematically searched for suitable studies from their inception to November 20, 2020. Language is limited with English and Chinese. Strategy will be built according to guidance from the Cochrane handbook. Search strategy for Excerpt Medical Database is shown in Table 1, and similar strategies will be modified and applied for other electronic databases.

**Table 1:** Search strategy performed in PubMed.

Number	Search items
1	Randomized controlled trial
2	Controlled clinical trial
3	Randomly
4	Randomized
5	Trail
6	Or/1–5
7	Tuina
8	Chinese tuina
9	Massage
10	Massage therapy
11	Chinese massage
12	Manipulation
13	Chinese manipulation therapy
14	Chinese manipulation
15	Or/7–14
16	asthma
17	Allergic asthma
18	Bronchial asthma
19	Or/16–18
20	6, 15, and 19

### 3.4 Study selection and management

**Figure 1:**
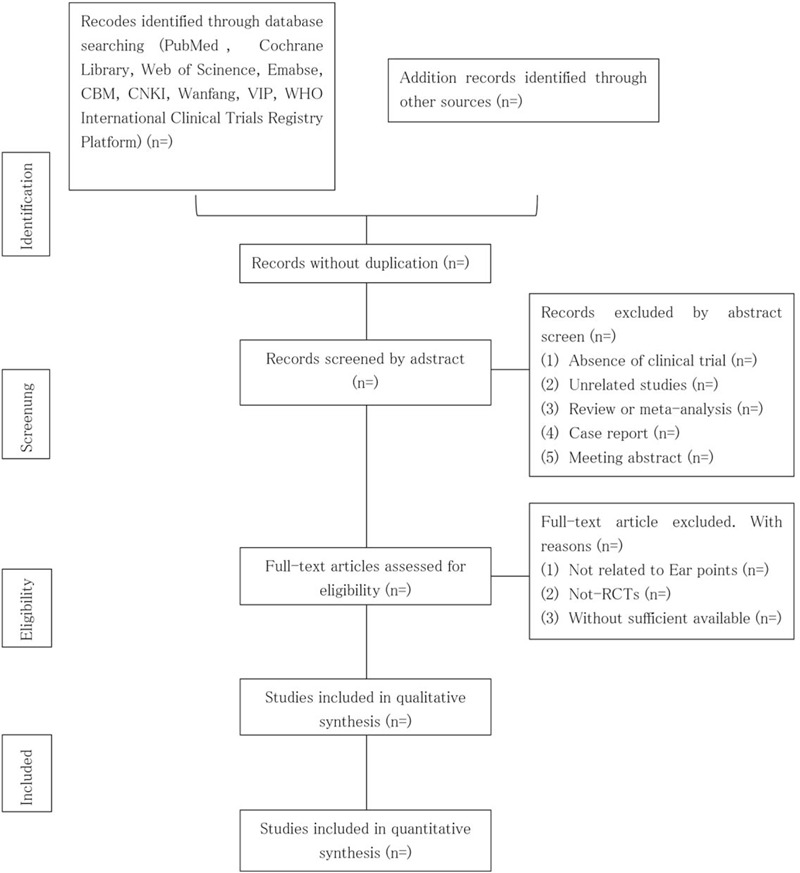
Flow diagram of studies selection process.

First, three review authors (CW, ZF, and MZ) will identify the titles and abstracts of retrieve dates independently. Endnote X9 software will be used for literature managing and records searching. Secondly, two review authors (YJ and ZW) will be reading the full text of preliminary selective articles and select suitable studies according to inclusion criteria. Finally, selecting articles will be put together. If there are some disagreements regarding inclusion and exclusion, we will make a group discussion. The details of whole selection procedure will be shown in a PRISMA follow diagram (Fig. 1)

### 3.5 Data extraction and management

Two reviewers (ZC and CW) will be charged of the data extraction according to the Cochrane Handbook for Systematic Reviews of Intervention. Information extraction form consisted mainly of following items:

1. Publication information (the first author, year, country);
2. Participants (sample size, source, age, gender, asthma types and severity, inclusion and exclusion criteria, etc);
3. Intervention (Tuina types, training frequencies and training time of every time, total training time, etc);
4. Comparison (other forms treatments, frequencies, treatment times);
5. Efficacy evaluation: Main observation indicators secondary observation indicators safety indicators and number of obverse reactions;
6. Outcomes (scale instruments, assessment time, results details, adverse event, cost and funding sources). Any disagreements will be resolved by discussion in group. The experience reviewer (YJ) will make the final decision.

### 3.6 Dealing with missing data

To some extent, the missing data have an influence on study result. When we make the decision of excluding, we need to contact the authors request the missing or incomplete data that will be further check and record. If relevant data are not exit or acquired, we will exclude them from analysis.

### 3.7 Quality assessment/risk of bias analysis

According to the guidance of the Cochrane Handbook for Systematic Review of Interventions, the quality of included RCTs will be assessed by 2 reviewers (YG and LC). The items of quality including inclusion criteria, sample size estimation, baseline, randomization, allocation sequence concealment, binding, selective reporting, missing data managements, and other bias. Evidence quality will be shown as high, unclear, low risk of bias in accordance with the criteria of the risk judgment. If there are disagreements, an experience researcher (YJ) will make the decision.

### 3.8 Strategy of data synthesis

The Revman 5.3 software provided by Cochrane Collaboration is used to perform all statistical analyses. All outcomes are presented as continuous variables in this review. We will perform a pairwise meta-analysis using a random-effect model. To determine the effect size, risk rations with 95% confidence intervals will be calculated for dichotomous outcomes and the standard mean difference with 95% confidence intervals will be calculated for continuous outcomes. Depending on the heterogeneity assessed by the *I*^2^ statistic, a fixed- ore random-effect model will be used. If there is statistical heterogeneity, sensitivity to explore the source of heterogeneity.

### 3.9 Publication bias analysis

If ten or more studies are in included in the meta-analysis, we will sight publication biases and poor methodological quality by funnel plots.

### 3.10 Assessment of heterogeneity

#### 3.10.1 Sensitivity analysis

For the quality analysis, we will conduct a sensitivity analysis of main outcomes to explore an individual study's influence of bias on results.

#### 3.10.2 Subgroup and meta-regression analysis

If the heterogeneity is apparent, subgroup and meta-regression analysis will be set up according to the characteristic of the study to explore the source of heterogeneity with regard to age, gender, region, type of control interventions, type of Tuina, and frequency and duration.

## 4 Discussion

As a chronic respiratory disease with a relatively high incidence in recent years, a large number of domestic and foreign scholars have conducted in-depth research on the etiology, clinical manifestations and treatment of asthma.^[13]^ Although asthma is as a chronic respiratory disease with a relatively high incidence in recent years, a large number of domestic and foreign scholars have conducted in-depth research on the etiology, clinical manifestations and treatment of asthma.^[14]^ Although asthma is currently a huge problem for medicine, the development of new drugs and new technologies has brought new expectations to asthma patients. Clinically, when selecting a treatment strategy, it is necessary to combine individual patient conditions and biomarkers to adopt personalized treatment. In recent years, the clinical RCT of asthma has been increasing, but it is still unsatisfactory in the diagnosis and treatment of diseases.^[15]^ Clinicians have not yet reached a consensus on the treatment principles and assessment of the disease, and lack uniform standardization standards.^[16]^ At present, there has not been a large-scale epidemiological investigation of the disease, and there are few reports in the literature. Chinese medicine has a profound theoretical foundation and rich clinical experience in the treatment of asthma. Tuina is an indispensable part of traditional Chinese medicine, with the characteristics of small side effects and easy operation.^[17]^ Due to the limited data collected, for example, some documents are still in the private sector and have not been published in the electronic database, this paper may have some limitations. This paper may be the first systematic review of the whole literature on the treatment of scapulohumeral periarthritis with Tuina. The aim is to make an objective, true and high-quality evaluation of the existing literature on clinical trials of Tuina for asthma at home and abroad.

## Author contributions

**Data curation:** Zhipeng Fan.

**Investigation:** Mao Zhao.

**Project administration:** Zhaoxing Chen.

**Resources:** Yuchang Jiang.

**Software:** Zhaodi Wang.

**Writing – original draft:** Changhong Wang.

**Writing – review & editing:** Yong Jiang.

## References

[1] Schatz M Rosenwasser L The allergic asthma phenotype. J Allergy Clin Immunol Pract 2014;2:645–8. 25439351 10.1016/j.jaip.2014.09.004

[2] Mastrorilli C Posa D Cipriani F Asthma and allergic rhinitis in childhood: what's new. Pediatr Allergy Immunol 2016;27:795–803. 27862336 10.1111/pai.12681

[3] Finotto S Resolution of allergic asthma. Semin Immunopathol 2019;41:665–74. 31705318 10.1007/s00281-019-00770-3

[4] Schoettler N Rodríguez E Weidinger S Advances in asthma and allergic disease genetics: Is bigger always better? J Allergy Clin Immunol 2019;144:1495–506. 31677964 10.1016/j.jaci.2019.10.023 PMC6900451

[5] Chung KF Clinical management of severe therapy-resistant asthma. Expert Rev Respir Med 2017;11:395–402. 28388228 10.1080/17476348.2017.1317597

[6] Tao WW Jiang H Tao XM Effects of acupuncture, Tuina, Tai Chi, Qigong, and traditional Chinese medicine five-element music therapy on symptom management and quality of life for cancer patients: a meta-analysis. J Pain Symptom Manage 2016;51:728–47. 26880252 10.1016/j.jpainsymman.2015.11.027

[7] Mo Z Li D Zhang R Comparisons of the effectiveness and safety of Tuina, acupuncture, traction, and Chinese herbs for lumbar disc herniation: a systematic review and network meta-analysis. Evid Based Complement Alternat Med 2019;2019:6821310. 31015852 10.1155/2019/6821310 PMC6446119

[8] Lu P Ye ZQ Qiu J Acupoint-tuina therapy promotes lactation in postpartum women with insufficient milk production who underwent caesarean sections. Medicine (Baltimore) 2019;98:e16456. 31464890 10.1097/MD.0000000000016456 PMC6736488

[9] Fan Z Tian Q Guo R Tuina for low back pain: protocol for a systematic review and meta-analysis. Medicine (Baltimore) 2018;97:e11979. 30142831 10.1097/MD.0000000000011979 PMC6113039

[10] Lu T Zhang H Yin L Chinese pediatric Tuina on children with acute diarrhea: study protocol for a randomized sham-controlled trial. Trials 2019;20:689. 31815655 10.1186/s13063-019-3818-1 PMC6902472

[11] Zhang Y Gao C Chen D Tuina massage improves cognitive functions of hypoxic-ischemic neonatal rats by regulating genome-wide DNA hydroxymethylation levels. Evid Based Complement Alternat Med 2019;2019:1282085. 31772590 10.1155/2019/1282085 PMC6854251

[12] Dong Y Zhao R Wang C Tuina for osteoporosis: a systematic review protocol. Medicine (Baltimore) 2018;97:e9974. 29465598 10.1097/MD.0000000000009974 PMC5841994

[13] Kwah JH Peters AT Asthma in adults: principles of treatment. Allergy Asthma Proc 2019;40:396–402. 31690379 10.2500/aap.2019.40.4256

[14] Kanagaratham C Radzioch D Allergic asthma: a summary from genetic basis, mouse studies, to diagnosis and treatment. Curr Pharm Des 2016;22:6261–72. 27573930 10.2174/1381612822666160829141708

[15] Nagarajan S Ahmad S Quinn M Allergic sensitization and clinical outcomes in urban children with asthma, 2013-2016. Allergy Asthma Proc 2018;39:281–8. 30095393 10.2500/aap.2018.39.4147 PMC6052173

[16] Casale TB Amin BV Allergic rhinitis/asthma interrelationships. Clin Rev Allergy Immunol 2001;21:27–49. 11471339 10.1385/CRIAI:21:1:27

[17] Rong J Feng H Li J Tuina for children with myopia: a protocol for a systematic review. Medicine (Baltimore) 2019;98:e18342. 31852130 10.1097/MD.0000000000018342 PMC6922427

